# Symmetry breaking and structure of a mixture of nematic liquid crystals and anisotropic nanoparticles

**DOI:** 10.3762/bjoc.6.74

**Published:** 2010-07-07

**Authors:** Marjan Krasna, Matej Cvetko, Milan Ambrožič

**Affiliations:** 1 University of Maribor, Faculty of Natural Sciences and Mathematics, Koroška cesta 160, 2000 Maribor, Slovenia; 2 University of Maribor, Faculty of Arts, Koroška cesta 160, 2000 Maribor, Slovenia; 3 Regional Development Agency Mura Ltd. Lendavska 5a, 9000 Murska Sobota, Slovenia

**Keywords:** liquid crystals, nanoparticles, orientational order, quenched disorder, symmetry breaking

## Abstract

Orientational ordering of a homogeneous mixture of uniaxial liquid crystalline (LC) molecules and magnetic nanoparticles (NPs) is studied using the Lebwohl–Lasher lattice model. We consider cases where NPs tend to be oriented perpendicularly to LC molecules due to elastic forces. We study domain-type configurations of ensembles, which are quenched from the isotropic phase. We show that for large enough concentrations of NPs the long range uniaxial nematic ordering is replaced by short range order exhibiting strong biaxiality. This suggests that the impact of NPs on orientational ordering of LCs for appropriate concentrations of NPs is reminiscent to the influence of quenched random fields which locally enforce a biaxial ordering.

## Introduction

The past decade has witnessed an increased interest in the study of two-component mixtures consisting of nanoparticles (NPs) in a host material [1–5]. A characteristic feature of a nanoparticle is that at least one of its dimensions is of the order of nanometers. Such systems are expected to play an important role in the emerging field of nanotechnology and also in composites with extraordinary material properties. These mixtures can, in general, exhibit properties which are not encountered in either of the isolated components, thus opening the door to new applications.

Of particular interest are cases where the host component is a soft material [6]. These materials can then exhibit relatively strong responses, even to local low-energy excitations. Typical representatives of soft materials, with great application potential, are various liquid crystals phases [6]. Their soft character is due to continuous symmetry breaking by which LC phases are reached, giving rise to Goldstone excitation modes. LCs are also optically anisotropic and transparent, whose structure can be readily controlled by the confining surfaces and by applying an external electric or magnetic field. LCs exhibit a rich pallet of different structures and phases that can display almost all physical phenomena. In addition, the chemistry of LCs is relatively well developed; therefore the synthesis of LC molecules with the desired behavior can be achieved with a certain degree of ease. As a result of these properties, even pure LC systems have found several applications, in particular in the electro-optics industry.

We henceforth limit our attention to rod-like LC molecules and to thermotropic LCs in which liquid crystal phases are induced by lowering the temperature from the ordinary liquid (isotropic) phase. The nematic configuration represents the simplest liquid crystal phase [6]. In the bulk nematic phase LC molecules tend to be oriented homogeneously along a single symmetry breaking direction. At the mesoscopic level the average local orientational ordering is commonly described by the nematic director field 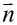. The directions ±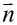 of this unit vector field are physically equivalent, reflecting the so called head-to tail invariance of LC phase on the mesoscopic scale.

If ensembles are suddenly quenched from the isotropic to the lower symmetry nematic phase, then unavoidably a domain pattern forms [7]. The reason behind this is continuous symmetry breaking and causality (i.e., the finite speed at which information spreads in a system). Generality of this mechanism gives rise to a broad universality of the phenomenon. The basic features of domain pattern dynamics in a pure bulk are described by the Kibble–Zurek mechanism [8–9] which was originally introduced to explain the formation of topological defects in the early universe following the big bang [8]. For the latter purposes, we summarize main features of this universal mechanism for the case of the isotropic–nematic (I–N) phase transition. In the I–N quench the continuous orientational symmetry is broken. A randomly chosen configuration of the symmetry breaking field 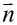 is established in causally disconnected parts [7]. This choice is based on local fluctuation mediated preferences. Consequently, a domain structure appears, which is well characterized by a single domain length *ξ*_d_. At the domain walls topological defects form. Such a structure is energetically costly due to the high concentration of domain walls and defects. The costs on average domain growth with time can be reduced by mutual annihilation of defects [10–11]. In the pure bulk system a spatially homogeneous structure is gradually attained. However, if impurities are present, they can pin the defects [12–14]. Consequently, the domain structure can be stabilized.

In this contribution we study numerically a mixture of uniaxial nematic liquid crystals and rod-like NPs using a Lebwohl–Lasher [15–16] lattice model. We consider cases where NPs and LC molecules tend to be oriented perpendicular to each other and show that in such systems NPs induce strong biaxiality [17] in LC ordering. Furthermore, we demonstate that NPs can stabilize the domain pattern giving rise to short range ordering in the nematic LC phase [18–19].

## Results and Discussion

### Model

The three-dimensional (3D) spin model simultaneously describes the orientational field of a LC *molecule* and the dimensionless magnetization of the magnetic component. We henceforth refer to these elements as *nematic spins* and *magnetic spins*, respectively. Here a *molecule* might represent a small group of real LC molecules. The system is represented by a rectangular simulation cell consisting a lattice of *N = N_x_* *× N_y_* *× N_z_* sites. Each site is enumerated by a set of indices (*i*, *j*, *k*), where 1 ≤ *i ≤ N_x_*, 1 *≤ j ≤ N_y_* and 1 *≤ k ≤ N_z_*, and is occupied either by nematic or magnetic spin 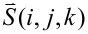 ≡ 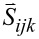, which may point in any direction. At the mesoscopic level, nematic spins represent the conventional nematic director field. Neighboring alike spins tend to align in parallel directions, whilst nematic and magnetic spins tend to be perpendicular to each other. The probability for a specific site to contain the magnetic component (instead of LC) is *x*, yielding on average *xN* magnetic spins in the cell. The parameter *x* is set in advance, and then the computer random generator is used to insert randomly magnetic spins into the cell according to the probability *x*. During the simulation (relaxation of spins approaching equilibrium) this positional configuration of magnetic and nematic spins remains unchanged.

The total energy of the system is given by:

[1]
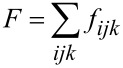


where the energy term *f_ijk_* equals:


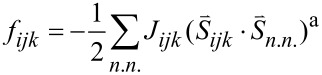


The six terms include the spin interactions between the nearest neighbors (denotation *n*.*n*.): the spin 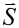
(*i*, *j*, *k*) interacts with 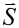
(*i*+1, *j*, *k*), 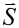
(*i*−1, *j*, *k*), 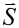
(*i*, *j*+1, *k*), 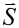
(*i*, *j*−1, *k*), 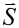
(*i*, *j*, *k*+1) and 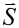
(*i*, *j*, *k*−1), respectively. The factor 1/2 is included because each neighboring spin pair is counted twice in the double sum. The interaction *J_ijk_* is equal to a constant, *J*_LC–LC_ or *J*_LC–NP_ or *J*_NP–NP_, reflecting the cases where an interacting pair is a LC–LC spin, LC–magnetic spin or magnetic–magnetic spin, respectively. We scale the system into a dimensionless form by setting *J*_LC–LC_ = 1. The parameter (*J*_NP–NP_) is taken as positive since neighbouring magnetic spins tend to align parallel. By contrast, we set *J*_LC–NP_ < 0, tending to orient LC molecules and NP perpendicularly. In the simulations we take *J*_LC–LC_ = *J*_NP–NP_ = 1, and *J*_LC–NP_ is either set to −1, −2 or −4. The exponent *a* is equal to 1 for magnetic–magnetic coupling while for nematic–nematic or nematic–magnetic coupling it has the value 2. The different values for exponent *a* for the different kinds of spins reflects their different symmetry properties. Unlike magnetic spins, nematic spins are insensitive to an inversion operation: 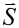 ≡ −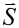.

The equilibrium spin configuration is obtained by minimizing the total interaction energy with respect to all the spins. Therefore, we neglect thermal fluctuations. Consequently, our approach is sensible, deep in the nematic LC phase region, i.e. well below the isotropic–nematic phase transition. In order to satisfy the normalization of the spin vectors, 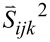 = 1, the “operational” total interaction energy must be rewritten as:

[2]
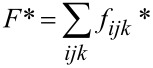


where:


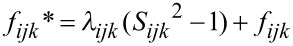


with Lagrange multipliers *λ_ijk_*, which must also be evaluated in order to solve the system.

From the obtained spin configurations, we calculate various quantities which reflect the structural properties of the system. One of these is the equilibrium total energy which is conveniently normalized to one spin site:

[3]
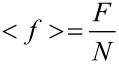


and represents the average energy term per spin.

The orientational ordering of the LC part of the system can be characterized by the traceless symmetric order parameter tensor with 3 × 3 components:

[4]
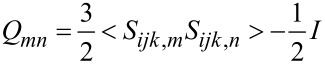


where *S_ijk,m_* is the *m*-th component of the LC spin 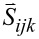. The brackets <...> denote the average of the quantity through the simulation cell and *I* is the identity matrix.

The degree of biaxiality of the LC component is measured with the *biaxiality parameter* [20–21]

[5]
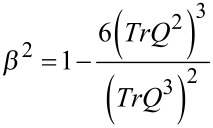


where 0 ≤ *β*^2^ ≤ 1. The uniaxial states are characterized by *β*^2^ = 0, and the states exhibiting maximal biaxiality by *β*^2^ = 1.

Average structural characteristics of the system can be inferred from the orientational correlation function:

[6]
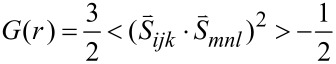


Here <...> denotes averaging over spin pairs separated by a distance *r*. Due to the isotropic character of our ensembles, the relationship 
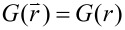
 holds.

The correlation function is calculated numerically in the following manner. First, the “vector index difference” (Δ*i*, Δ*j*, Δ*k*) is chosen, for instance (2, 1, −3), giving the vector relative position of correlated spin pairs in units of the nearest neighbor distance *a*_0_. Next, the pairs *r* and *G*, corresponding to (Δ*i*, Δ*j*, Δ*k*), are calculated:


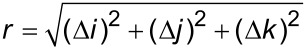



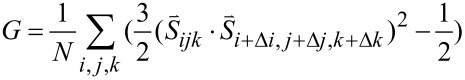


Averaging of individual pair correlations over the spin lattice is used. To avoid technical difficulties, periodic boundary conditions are performed when one of the indices exceeds the limit. For example, if *i* = *N_x_* and Δ*i* = 3, we take *i* + Δ*i* = 3 instead of *N_x_* + 3. This is in accordance with the periodic boundary condition used in the evaluation of the spins themselves (for instance, in Equation 2 the “right” nearest neighbor of the spin on the right border of simulation cell with index *i* = *N_x_* has the corresponding index *i* = 1).

The “vector index difference” (Δ*i*, Δ*j*, Δ*k*) is systematically varied to obtain the *G*(*r*) dependence, and the data pairs (*r*, *G*) are sorted by increasing distance *r*. However, the same *r* may correspond to different combinations of index differences (Δ*i*, Δ*j*, Δ*k*), for instance in all sign combinations of (±Δ*i*, ±Δ*j*, ±Δ*k*). By inspecting of the results of numerical simulations, we find that *G* is indeed equal in cases with the same *r*, except for small unimportant statistical variations which are subsequently annulled by averaging *G* for data pairs (*r*, *G*) with the same *r*.

To extract structural details from a calculated *G*(*r*) dependence, we fit it with the ansatz [22]

[7]
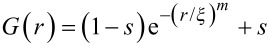


where *ξ*, *m* and *s* are adjustable parameters. Distances are scaled with respect to the nearest neighbour distance *a*_0_. The *nematic domain length ξ* estimates the average length over which LC molecules are relatively well correlated. The distribution width of *ξ* values is measured by the *domain dispersion parameter m*. Dominance of a single coherence length in the system is signalled by *m* ≈ 1. A magnitude and system size dependence of the *range parameter s* reveals the degree of ordering within the system. The case *s* = 0 indicates short range ordering (SRO), whilst a finite value of *s* is consistent with either long range ordering (LRO) or quasi long range ordering (QLRO). To distinguish between these two cases a finite size analysis *s*(*N*) must be carried out. If *s*(*N*) saturates at a finite value, the system exhibits LRO. If *s*(*N*) dependence exhibits algebraic dependence on *N* then the system possesses QLRO. In our study the correlation function was calculated only for the LC component of the system.

### Results

We consider homogeneous mixtures of nematic LCs (*nematic spins*) and elongated NPs (*magnetic spins*). For sufficiently large concentrations *x* of NPs, such a system could undergo phase separation. In order to estimate roughly concentrations of NPs which are well soluble in a LC solvent, we focus on the chemical potential *μ* of NPs in the mixture. It can be expressed as [23]

[8]
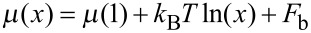


where *x* = *N*_NP_/(*N*_LC_ + *N*_NP_), *N*_NP_ (i.e., *N*_LC_) represents the number of NPs (i.e., LC molecules) in the system, *μ*(1) is the chemical potential in the solid phase, *k*_B_ is the Boltzmann constant, *T* is the temperature and *F*_b_ is the average binding energy of a NP with its surroundings. We further assume that the chemical potential in a diluted and solid NP state are comparable (depending on the chemical composition of both phases) and consequently, the system does not possess a tendency for phase separation. With this in mind we obtain *k*_B_*T* ln(*x*) + *F*_b_ ≈ 0. From this expression we get an estimate for the upper concentration *x*_max_ of NPs for which a homogeneous distribution is preserved:

[9]
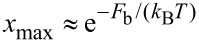


Therefore, high solubility is preferred by low binding energies and high temperatures. In order to discern the influence of geometrical details of NPs we consider dilute mixtures, where


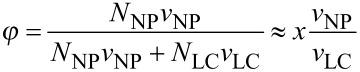


holds, where *φ* is the volume fraction of NPs and *v*_NP_ (i.e., *v*_LC_) is the volume of an average nanoparticle (i.e., LC molecule). Therefore, the upper volume fraction *φ*_max_ of NPs in a homogeneous mixture can be expressed as

[10]
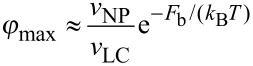


We next consider a mixture of a nematic LC phase and ferromagnetic NPs. Such mixtures are of interest for the development of LC materials with pronounced magnetic properties. It was shown [24] that in such materials orientational ordering is predominantly influenced by elastic interactions, which are several orders of magnitude greater than magnetic interactions. To demonstrate that we estimate at the mesoscopic level the typical energy changes related to the reorientation of the nematic director 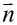 from the direction along the local effective magnetic field 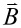 towards the perpendicular direction. Here we assume that 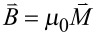 originates from magnetic NPs, where *μ*_0_ is the magnetic permittivity constant and 
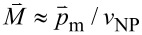
 represents the magnetization of NPs due to the magnetic dipole moment 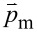. The resulting quadrupolar magnetic field free energy density change Δ*f*_B_ is approximately given by


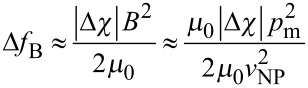


where Δ*χ* is the magnetic anisotropy of LC molecules [6] (which can be either positive or negative). Furthermore, introducing an elongated NP of length *d* into a LC, which via surface anchoring enforces elastic distortion in LC medium, typically gives rise to free energy penalties of the order Δ*F_e_* ≈ *Kd*, where *K* is the characteristic Frank nematic elastic constant. Typical nematic material constants are approximately given by *K* ≈ 10^−12^ J/m, |Δ*χ*| ≈ 10^−10^, and for rod like NPs of radius *r* = 1 nm we set *d*/*r* = 10 and *p_m_* ≈ *emu*. From this choice of parameters we obtain Δ*F_e_* >> eV, *v*_NP_Δ*f*_B_ << eV and consequently, *v*_NP_Δ*f*_B_/Δ*F_e_* << 1. Therefore, elastic forces predominantly influence orientational ordering of LC molecules that are surrounded by magnetic NPs.

Consequently, we henceforth limit the discussion to elastic interactions between LCs and NPs. In order to obtain qualitatively new features, we consider the case where NP and LC molecules tend to orient themselves perpendicular to each other. Such conditions are often encountered in other studies of mixtures of nematic LCs and magnetic inclusions reported in the literature [25–27]. On the other hand, we set it that isolated components possess the tendency for parallel orientation. We study structural and phase properties as a function of concentration of NPs in the diluted regime (i.e., *x* << 1) and of the interaction strength |*J*_LC–NP_| between NP and LC molecules. We monitor quasi stable nematic configurations after quenching the system from the isotropic phase.

**Figure 1:**
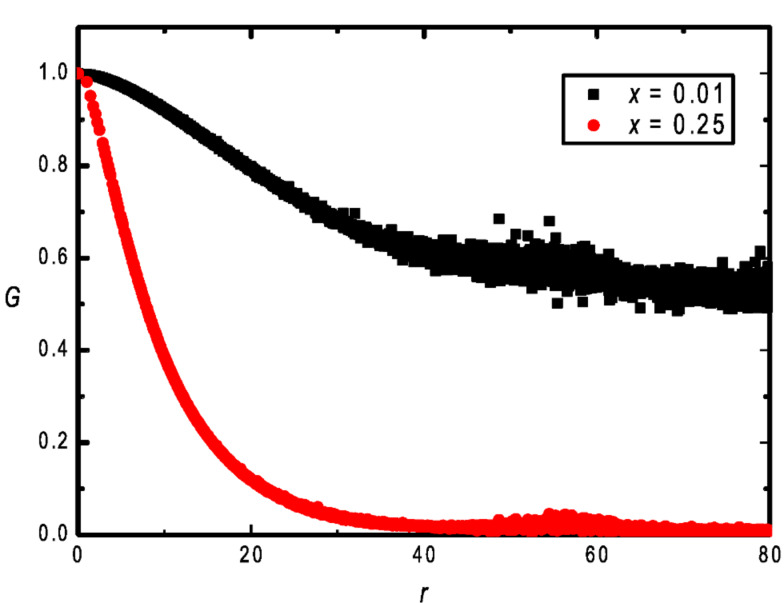
Nematic orientational correlation function *G*(*r*) for different values of *x* (*x* = 0.01 and 0.25), *N* = 80 × 80 × 80, *J*_LC–NP_ = −1, *J*_LC–LC_ = *J*_NP–NP_ = 1.

In Figure 1 we plot the correlation function *G*(*r*) measuring the degree of orientational order of LC molecules for different concentrations *x*. One sees that for sufficiently low concentrations the nematic long range order is preserved, which is manifested in a finite value of *s*. However, with increasing *x* the value of *s* decreases monotonously. Our numerical simulations suggest, that above a threshold value *x = x*_c_ long range ordering is replaced by short range ordering. Therefore, for a sufficiently large concentration the degree of disorder introduced by randomly distributed elongated nanoparticles is large enough to destroy the LRO favoured by the pure LC component.

**Figure 2:**
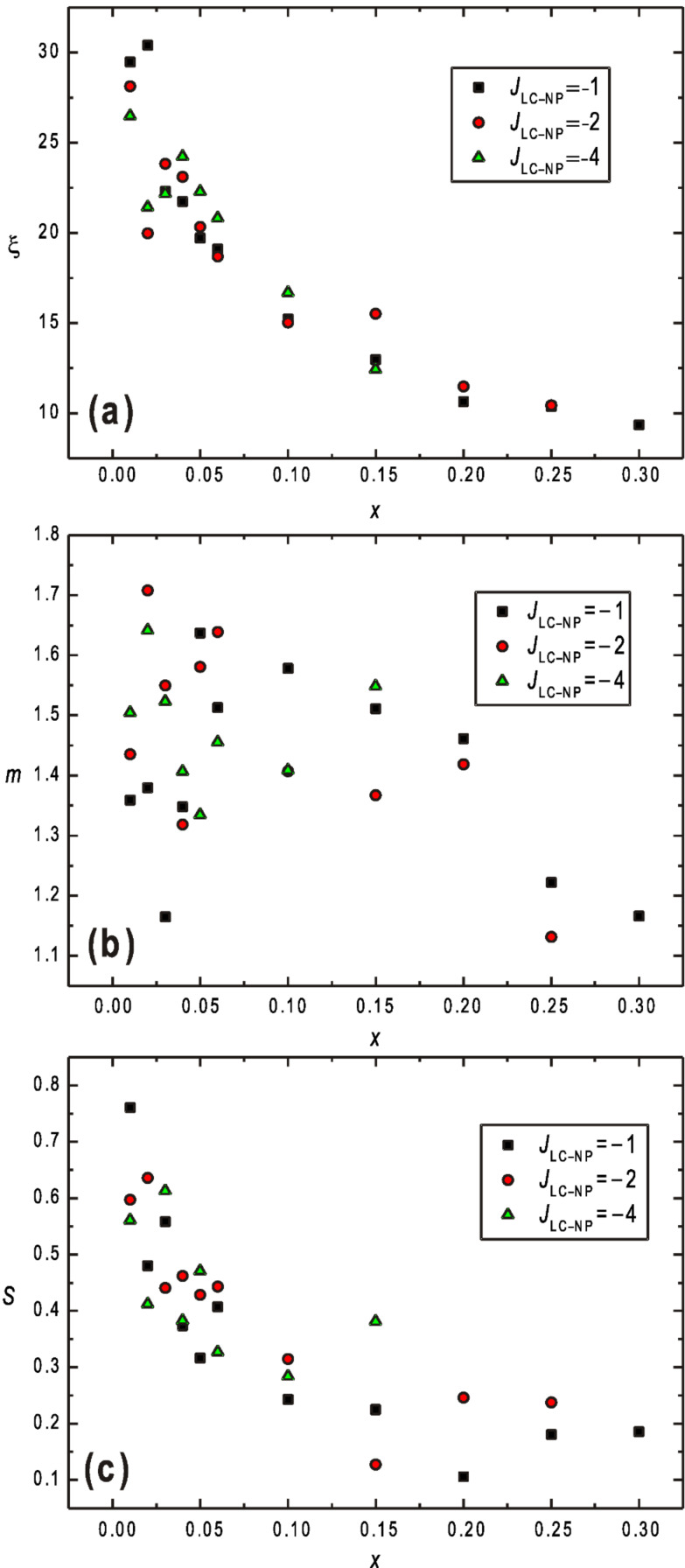
(a) The *nematic domain length ξ*, (b) the *domain dispersion parameter m*, and (c) the *range parameter s* as a function of *x* for different values of *J*_LC–NP_: −1, −2, and −4; *J*_LC–LC_ = *J*_LC–NP_ = 1, *N* = 80 × 80 × 80.

More details of this phenomenon are presented in Figure 2 where the average behaviour of systems, extracted by fitting Equation 7, is shown on increasing *x* for different interaction strengths |*J*_LC–NP_|. We plotted the *nematic domain length ξ* (Figure 2a), the *domain dispersion parameter m* (Figure 2b), and the *range parameter s* (Figure 2c). As intuitively expected, *ξ* decreases monotonously with *x*, see Figure 2a. Our simulations suggest 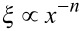, where *n* = 0.33 ± 0.03. The values of *m* are strongly scattered around the average value 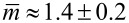, where a systematic trend on varying *x* is not observed as it is evident from Figure 2b. On the other hand, Figure 2c yields strong evidence that the LRO (or QLRO) is destroyed above a critical value *x*_c_. For *s*|*J*_LC–NP_| = 1 we obtain *x*_c_ ≈ 0.1. To test the existence of LRO we performed finite size analysis for *x = x*_c_/2 ≈ 0.05. Our numerical results do not show any systematic decrease of *s* on increasing 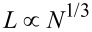. This suggests that the systems exhibit LRO if *x* <≈ *x*_c_.

**Figure 3:**
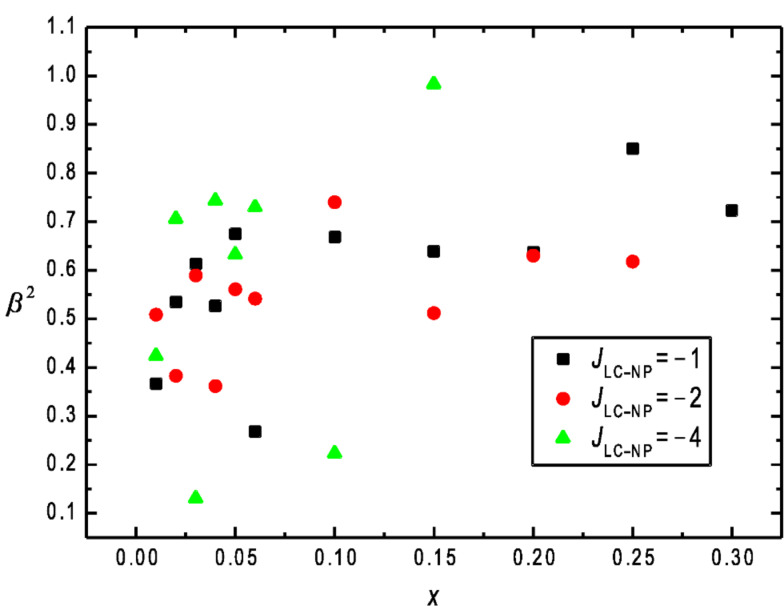
Degree of biaxiality *β*^2^ as a function of *x*; *J*_LC–NP_: −1, −2, and −4; *J*_LC–LC_ = *J*_LC–NP_ = 1, *N* = 80 × 80 × 80.

We next focus on degree of biaxiality within the ensembles studied. For this purpose we calculate the biaxiality parameter *β*^2^ as a function of *x* (Figure 3). It can be seen that the degree of biaxiality is surprisingly strong, even for relatively low values of *x*. The average degree of biaxiality *β*^2^ is larger than 0.5 above *x* = 0.05, which is surprisingly large. The reason behind this is the local tendency of NPs to reorient LC molecules perpendicular to them. This tendency is similar to that of an external electric or magnetic field acting on LCs with negative field anisotropy which tends to orient LC molecules perpendicularly to the field direction [6,28]. In such LC materials an external field imposes a finite degree of biaxiality.

## Conclusion

We studied numerically structural characteristics of a diluted mixture of nematic liquid crystals and ferromagnetic nanoparticles. The concentration *x* of NPs is low enough in order to avoid a phase separation process [29]. We consider cases where both LC molecules and NPs are elongated and tend to be oriented perpendicularly to each other [25–27]. For simulation we use a Lebwohl–Lasher lattice type model [15]. LC molecules and NPs tend to orient perpendicularly to each other. In actual samples such conditions could be realized for so called homeotropic [6] surface anchoring at the NP–LC interface of elongated NPs providing that *Wd*/*K* > 1. Here *W* is the anchoring strength, *K* is the characteristic nematic elastic constant and *d* is the length of a nanoparticle. We typically consider ensembles of *N* = 80 × 80 × 80 elements (i.e., LC molecules and NPs). In simulations we quench the systems from an isotropic phase, where orientations of all particles are randomly distributed.

Our simulations reveal that NPs act effectively as nematic domain pinning centres [30]. After quenching, nematic domains form due to continuous symmetry breaking. In a bulk system the domains would gradually grow in order to get rid of energetically expensive domain walls [7]. However, the presence of NPs stabilizes the domain pattern. We find that the average domain walls scales as 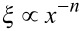, where *n* = 0.33 ± 0.03. Furthermore, for sufficiently large concentrations (*x*_c_ ≈ 0.1) the LRO (or QLRO) appears to be replaced by SRO. Our results also show that NPs strongly support biaxial states [17,31]. Even at relatively low concentrations the degree of biaxiality is surprisingly high. We obtain *β*^2^ > 0.5 above *x* ≈ 0.05.

One of calculated LC (blue) and NP (red) spin patterns in three perpendicular planes (*x*−*y*, *y*−*z*, and *x*−*z*) cutting the centre of simulation cube cell is presented in the graphical abstract. The lengths of lines representing individual spins in the pattern vary because one of spin components is perpendicular to the plane of view.
